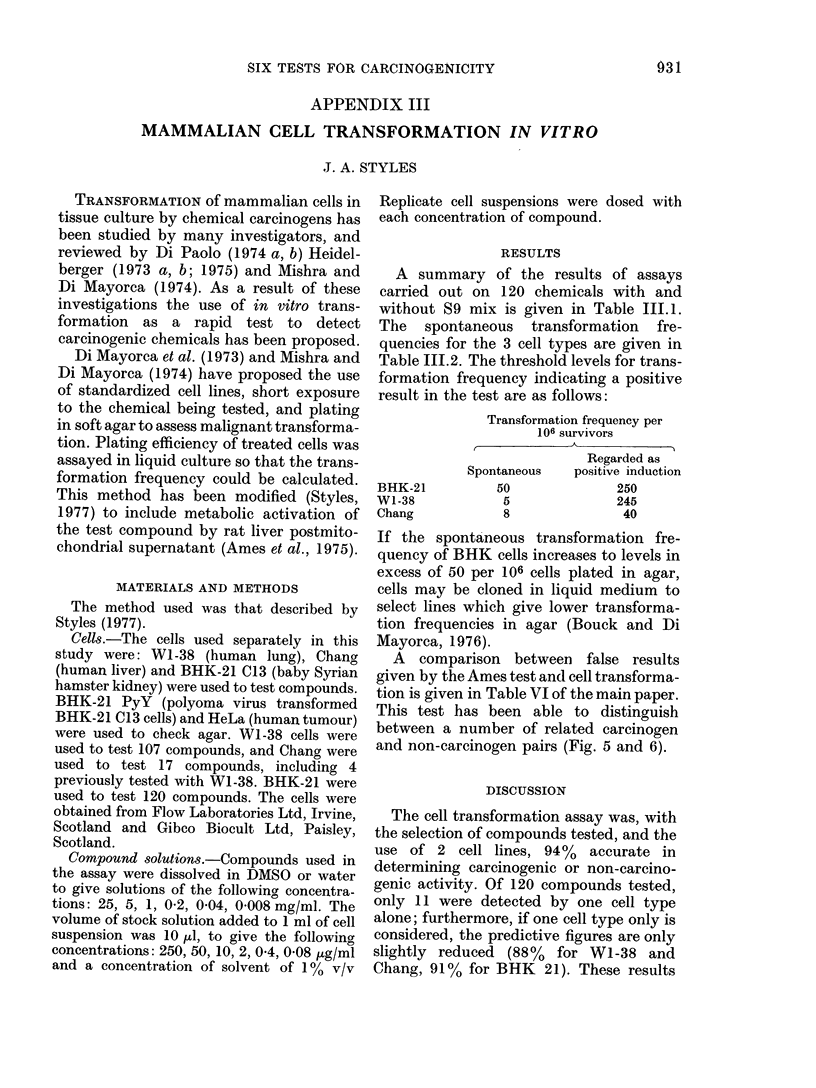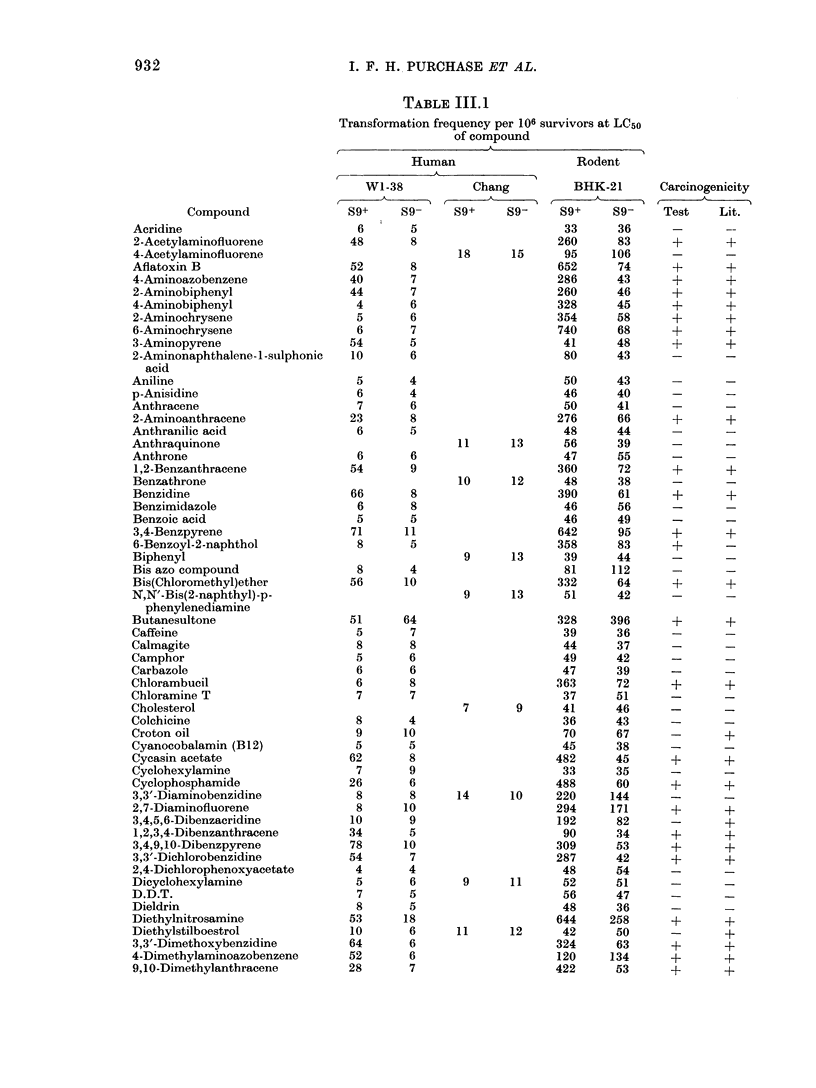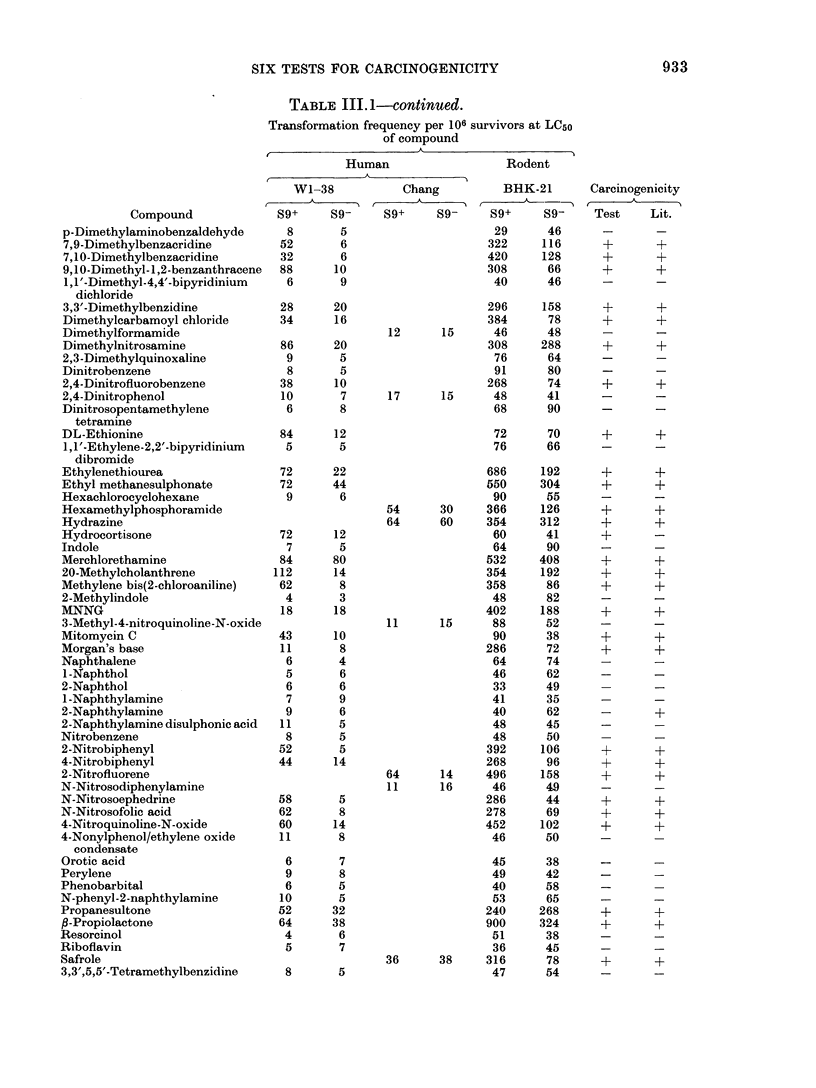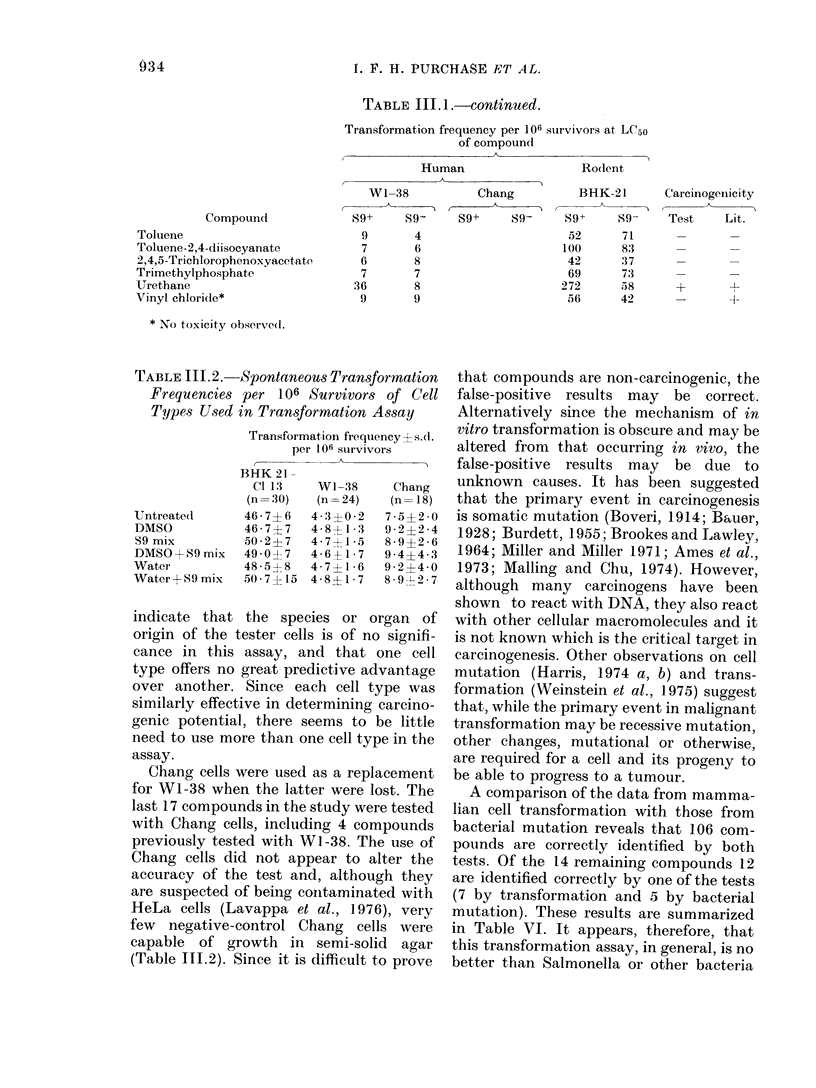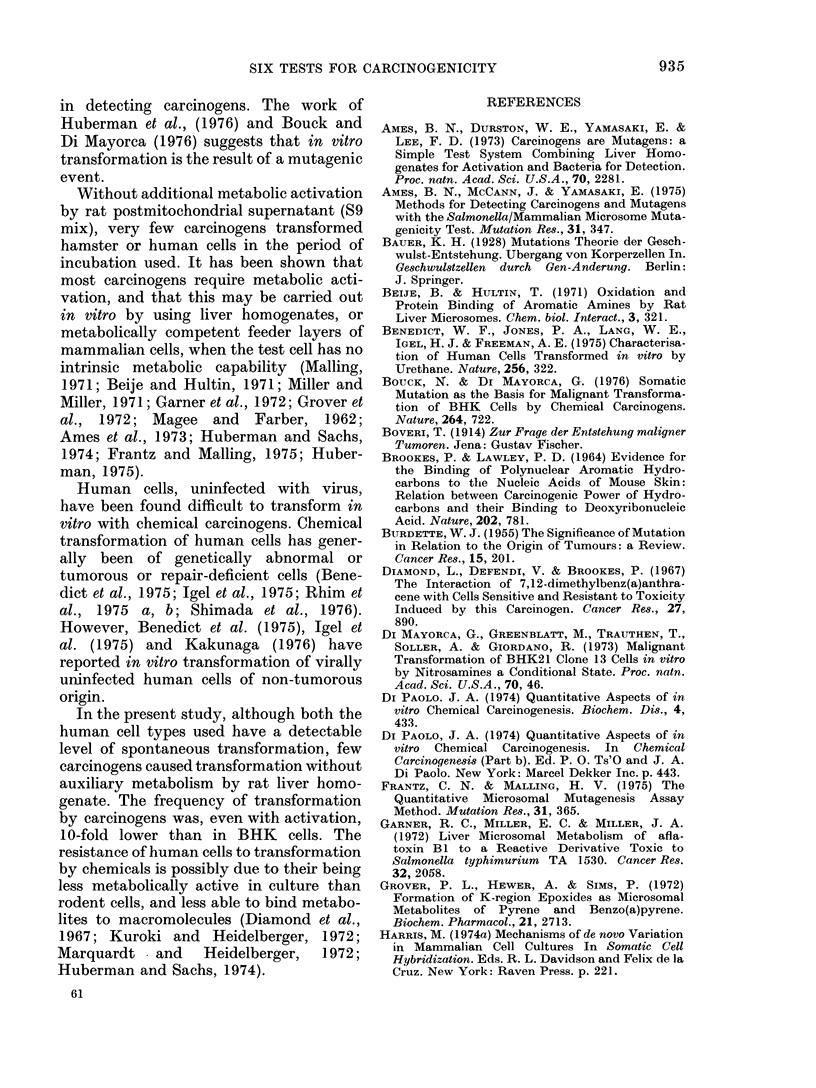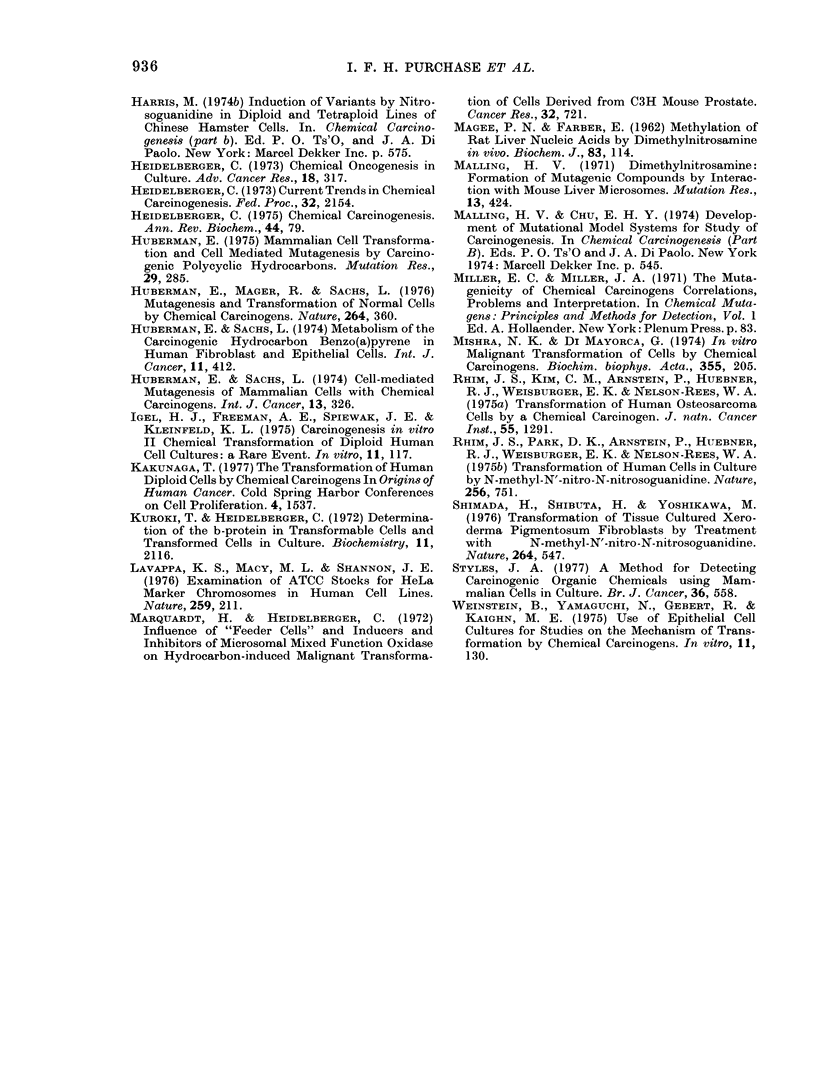# Mammalian cell transformation in vitro. Six tests for carcinogenicity.

**DOI:** 10.1038/bjc.1978.135

**Published:** 1978-06

**Authors:** J. A. Styles


### APPENDIX III

### MAMMALIAN CELL TRANSFORMATION *IN VITRO*

J. A. STYLES

TRANSFORMATION of mammalian cells in tissue culture by chemical carcinogens has been studied by many investigators, and reviewed by Di Paolo (1974 *a*, *b*) Heidelberger (1973 *a*, *b*; 1975) and Mishra and Di Mayorca (1974). As a result of these investigations the use of *in vitro* transformation as a rapid test to detect carcinogenic chemicals has been proposed.

Di Mayorca *et al.* (1973) and Mishra and Di Mayorca (1974) have proposed the use of standardized cell lines, short exposure to the chemical being tested, and plating in soft agar to assess malignant transformation. Plating efficiency of treated cells was assayed in liquid culture so that the transformation frequency could be calculated. This method has been modified (Styles, 1977) to include metabolic activation of the test compound by rat liver postmitochondrial supernatant (Ames *et al.*, 1975).

### MATERIALS AND METHODS

The method used was that described by Styles (1977).

*Cells.*—The cells used separately in this study were: W1-38 (human lung), Chang (human liver) and BHK-21 C13 (baby Syrian hamster kidney) were used to test compounds. BHK-21 PyY (polyoma virus transformed BHK-21 C13 cells) and HeLa (human tumour) were used to check agar. W1-38 cells were used to test 107 compounds, and Chang were used to test 17 compounds, including 4 previously tested with W1-38. BHK-21 were used to test 120 compounds. The cells were obtained from Flow Laboratories Ltd, Irvine, Scotland and Gibco Biocult Ltd, Paisley, Scotland.

*Compound solutions.*—Compounds used in the assay were dissolved in DMSO or water to give solutions of the following concentrations: 25, 5, 1, 0·2, 0·04, 0·008 mg/ml. The volume of stock solution added to 1 ml of cell suspension was 10 $\mu$l, to give the following concentrations: 250, 50, 10, 2, 0·4, 0·08 $\mu$g/ml and a concentration of solvent of 1% v/v Replicate cell suspensions were dosed with each concentration of compound.

### RESULTS

A summary of the results of assays carried out on 120 chemicals with and without S9 mix is given in Table III.1. The spontaneous transformation frequencies for the 3 cell types are given in Table III.2. The threshold levels for transformation frequency indicating a positive result in the test are as follows:

| | Transformation frequency per $10^6$ survivors | |
|---|---|---|
| | Spontaneous | Regarded as positive induction |
| BHK-21 | 50 | 250 |
| W1-38 | 5 | 245 |
| Chang | 8 | 40 |

If the spontaneous transformation frequency of BHK cells increases to levels in excess of 50 per $10^6$ cells plated in agar, cells may be cloned in liquid medium to select lines which give lower transformation frequencies in agar (Bouck and Di Mayorca, 1976).

A comparison between false results given by the Ames test and cell transformation is given in Table VI of the main paper. This test has been able to distinguish between a number of related carcinogen and non-carcinogen pairs (Fig. 5 and 6).

### DISCUSSION

The cell transformation assay was, with the selection of compounds tested, and the use of 2 cell lines, 94% accurate in determining carcinogenic or non-carcinogenic activity. Of 120 compounds tested, only 11 were detected by one cell type alone; furthermore, if one cell type only is considered, the predictive figures are only slightly reduced (88% for W1-38 and Chang, 91% for BHK 21). These results

TABLE III.1

| Compound | Transformation frequency per $10^6$ survivors at $LC_{50}$ of compound | | | | | | Carcinogenicity | |
|---|---|---|---|---|---|---|---|---|
| | Human | | | | Rodent | | | |
| | W1-38 | | Chang | | BHK-21 | | | |
| | S9+ | S9− | S9+ | S9− | S9+ | S9− | Test | Lit. |
| Acridine | 6 | 5 | | | 33 | 36 | − | − |
| 2-Acetylaminofluorene | 48 | 8 | | | 260 | 83 | + | + |
| 4-Acetylaminofluorene | | | 18 | 15 | 95 | 106 | − | − |
| Aflatoxin B | 52 | 8 | | | 652 | 74 | + | + |
| 4-Aminoazobenzene | 40 | 7 | | | 286 | 43 | + | + |
| 2-Aminobiphenyl | 44 | 7 | | | 260 | 46 | + | + |
| 4-Aminobiphenyl | 4 | 6 | | | 328 | 45 | + | + |
| 2-Aminochrysene | 5 | 6 | | | 354 | 58 | + | + |
| 6-Aminochrysene | 6 | 7 | | | 740 | 68 | + | + |
| 3-Aminopyrene | 54 | 5 | | | 41 | 48 | + | + |
| 2-Aminonaphthalene-1-sulphonic acid | 10 | 6 | | | 80 | 43 | − | − |
| Aniline | 5 | 4 | | | 50 | 43 | − | − |
| p-Anisidine | 6 | 4 | | | 46 | 40 | − | − |
| Anthracene | 7 | 6 | | | 50 | 41 | − | − |
| 2-Aminoanthracene | 23 | 8 | | | 276 | 66 | + | + |
| Anthranilic acid | 6 | 5 | | | 48 | 44 | − | − |
| Anthraquinone | | | 11 | 13 | 56 | 39 | − | − |
| Anthrone | 6 | 6 | | | 47 | 55 | − | − |
| 1,2-Benzanthracene | 54 | 9 | | | 360 | 72 | + | + |
| Benzathrone | | | 10 | 12 | 48 | 38 | − | − |
| Benzidine | 66 | 8 | | | 390 | 61 | + | + |
| Benzimidazole | 6 | 8 | | | 46 | 56 | − | − |
| Benzoic acid | 5 | 5 | | | 46 | 49 | − | − |
| 3,4-Benzpyrene | 71 | 11 | | | 642 | 95 | + | + |
| 6-Benzoyl-2-naphthol | 8 | 5 | | | 358 | 83 | + | − |
| Biphenyl | | | 9 | 13 | 39 | 44 | − | − |
| Bis azo compound | 8 | 4 | | | 81 | 112 | − | − |
| Bis(Chloromethyl)ether | 56 | 10 | | | 332 | 64 | + | + |
| N,N′-Bis(2-naphthyl)-p-phenylenediamine | | | 9 | 13 | 51 | 42 | − | − |
| Butanesultone | 51 | 64 | | | 328 | 396 | + | + |
| Caffeine | 5 | 7 | | | 39 | 36 | − | − |
| Calmagite | 8 | 8 | | | 44 | 37 | − | − |
| Camphor | 5 | 6 | | | 49 | 42 | − | − |
| Carbazole | 6 | 6 | | | 47 | 39 | − | − |
| Chlorambucil | 6 | 8 | | | 363 | 72 | + | + |
| Chloramine T | 7 | 7 | | | 37 | 51 | − | − |
| Cholesterol | | | 7 | 9 | 41 | 46 | − | − |
| Colchicine | 8 | 4 | | | 36 | 43 | − | − |
| Croton oil | 9 | 10 | | | 70 | 67 | − | + |
| Cyanocobalamin (B12) | 5 | 5 | | | 45 | 38 | − | − |
| Cycasin acetate | 62 | 8 | | | 482 | 45 | + | + |
| Cyclohexylamine | 7 | 9 | | | 33 | 35 | − | − |
| Cyclophosphamide | 26 | 6 | | | 488 | 60 | + | + |
| 3,3′-Diaminobenzidine | 8 | 8 | 14 | 10 | 220 | 144 | − | − |
| 2,7-Diaminofluorene | 8 | 10 | | | 294 | 171 | + | + |
| 3,4,5,6-Dibenzacridine | 10 | 9 | | | 192 | 82 | − | + |
| 1,2,3,4-Dibenzanthracene | 34 | 5 | | | 90 | 34 | + | + |
| 3,4,9,10-Dibenzpyrene | 78 | 10 | | | 309 | 53 | + | + |
| 3,3′-Dichlorobenzidine | 54 | 7 | | | 287 | 42 | + | + |
| 2,4-Dichlorophenoxyacetate | 4 | 4 | | | 48 | 54 | − | − |
| Dicyclohexylamine | 5 | 6 | 9 | 11 | 52 | 51 | − | − |
| D.D.T. | 7 | 5 | | | 56 | 47 | − | − |
| Dieldrin | 8 | 5 | | | 48 | 36 | − | − |
| Diethylnitrosamine | 53 | 18 | | | 644 | 258 | + | + |
| Diethylstilboestrol | 10 | 6 | 11 | 12 | 42 | 50 | − | + |
| 3,3′-Dimethoxybenzidine | 64 | 6 | | | 324 | 63 | + | + |
| 4-Dimethylaminoazobenzene | 52 | 6 | | | 120 | 134 | + | + |
| 9,10-Dimethylanthracene | 28 | 7 | | | 422 | 53 | + | + |

TABLE III.1—*continued.*

| Compound | Transformation frequency per $10^6$ survivors at $LC_{50}$ of compound: Human: W1–38 S9+ | W1–38 S9− | Chang S9+ | Chang S9− | Rodent: BHK-21 S9+ | BHK-21 S9− | Carcinogenicity: Test | Carcinogenicity: Lit. |
|---|---|---|---|---|---|---|---|---|
| p-Dimethylaminobenzaldehyde | 8 | 5 | | | 29 | 46 | − | − |
| 7,9-Dimethylbenzacridine | 52 | 6 | | | 322 | 116 | + | + |
| 7,10-Dimethylbenzacridine | 32 | 6 | | | 420 | 128 | + | + |
| 9,10-Dimethyl-1,2-benzanthracene | 88 | 10 | | | 308 | 66 | + | + |
| 1,1′-Dimethyl-4,4′-bipyridinium dichloride | 6 | 9 | | | 40 | 46 | − | − |
| 3,3′-Dimethylbenzidine | 28 | 20 | | | 296 | 158 | + | + |
| Dimethylcarbamoyl chloride | 34 | 16 | | | 384 | 78 | + | + |
| Dimethylformamide | | | 12 | 15 | 46 | 48 | − | − |
| Dimethylnitrosamine | 86 | 20 | | | 308 | 288 | + | + |
| 2,3-Dimethylquinoxaline | 9 | 5 | | | 76 | 64 | − | − |
| Dinitrobenzene | 8 | 5 | | | 91 | 80 | − | − |
| 2,4-Dinitrofluorobenzene | 38 | 10 | | | 268 | 74 | + | + |
| 2,4-Dinitrophenol | 10 | 7 | 17 | 15 | 48 | 41 | − | − |
| Dinitrosopentamethylene tetramine | 6 | 8 | | | 68 | 90 | − | − |
| DL-Ethionine | 84 | 12 | | | 72 | 70 | + | + |
| 1,1′-Ethylene-2,2′-bipyridinium dibromide | 5 | 5 | | | 76 | 66 | − | − |
| Ethylenethiourea | 72 | 22 | | | 686 | 192 | + | + |
| Ethyl methanesulphonate | 72 | 44 | | | 550 | 304 | + | + |
| Hexachlorocyclohexane | 9 | 6 | | | 90 | 55 | − | − |
| Hexamethylphosphoramide | | | 54 | 30 | 366 | 126 | + | + |
| Hydrazine | | | 64 | 60 | 354 | 312 | + | + |
| Hydrocortisone | 72 | 12 | | | 60 | 41 | + | − |
| Indole | 7 | 5 | | | 64 | 90 | − | − |
| Merchlorethamine | 84 | 80 | | | 532 | 408 | + | + |
| 20-Methylcholanthrene | 112 | 14 | | | 354 | 192 | + | + |
| Methylene bis(2-chloroaniline) | 62 | 8 | | | 358 | 86 | + | + |
| 2-Methylindole | 4 | 3 | | | 48 | 82 | − | − |
| MNNG | 18 | 18 | | | 402 | 188 | + | + |
| 3-Methyl-4-nitroquinoline-N-oxide | | | 11 | 15 | 88 | 52 | − | − |
| Mitomycin C | 43 | 10 | | | 90 | 38 | + | + |
| Morgan's base | 11 | 8 | | | 286 | 72 | + | + |
| Naphthalene | 6 | 4 | | | 64 | 74 | − | − |
| 1-Naphthol | 5 | 6 | | | 46 | 62 | − | − |
| 2-Naphthol | 6 | 6 | | | 33 | 49 | − | − |
| 1-Naphthylamine | 7 | 9 | | | 41 | 35 | − | − |
| 2-Naphthylamine | 9 | 6 | | | 40 | 62 | − | + |
| 2-Naphthylamine disulphonic acid | 11 | 5 | | | 48 | 45 | − | − |
| Nitrobenzene | 8 | 5 | | | 48 | 50 | − | − |
| 2-Nitrobiphenyl | 52 | 5 | | | 392 | 106 | + | + |
| 4-Nitrobiphenyl | 44 | 14 | | | 268 | 96 | + | + |
| 2-Nitrofluorene | | | 64 | 14 | 496 | 158 | + | + |
| N-Nitrosodiphenylamine | | | 11 | 16 | 46 | 49 | − | − |
| N-Nitrosoephedrine | 58 | 5 | | | 286 | 44 | + | + |
| N-Nitrosofolic acid | 62 | 8 | | | 278 | 69 | + | + |
| 4-Nitroquinoline-N-oxide | 60 | 14 | | | 452 | 102 | + | + |
| 4-Nonylphenol/ethylene oxide condensate | 11 | 8 | | | 46 | 50 | − | − |
| Orotic acid | 6 | 7 | | | 45 | 38 | − | − |
| Perylene | 9 | 8 | | | 49 | 42 | − | − |
| Phenobarbital | 6 | 5 | | | 40 | 58 | − | − |
| N-phenyl-2-naphthylamine | 10 | 5 | | | 53 | 65 | − | − |
| Propanesultone | 52 | 32 | | | 240 | 268 | + | + |
| β-Propiolactone | 64 | 38 | | | 900 | 324 | + | + |
| Resorcinol | 4 | 6 | | | 51 | 38 | − | − |
| Riboflavin | 5 | 7 | | | 36 | 45 | − | − |
| Safrole | | | 36 | 38 | 316 | 78 | + | + |
| 3,3′,5,5′-Tetramethylbenzidine | 8 | 5 | | | 47 | 54 | − | − |

TABLE III.1.—*continued.*

| Compound | Transformation frequency per $10^6$ survivors at $LC_{50}$ of compound: Human: W1–38 S9+ | W1–38 S9− | Chang S9+ | Chang S9− | Rodent: BHK-21 S9+ | BHK-21 S9− | Carcinogenicity Test | Carcinogenicity Lit. |
|---|---|---|---|---|---|---|---|---|
| Toluene | 9 | 4 | | | 52 | 71 | − | − |
| Toluene-2,4-diisocyanate | 7 | 6 | | | 100 | 83 | − | − |
| 2,4,5-Trichlorophenoxyacetate | 6 | 8 | | | 42 | 37 | − | − |
| Trimethylphosphate | 7 | 7 | | | 69 | 73 | − | − |
| Urethane | 36 | 8 | | | 272 | 58 | + | + |
| Vinyl chloride* | 9 | 9 | | | 56 | 42 | − | + |

* No toxicity observed.

TABLE III.2.—*Spontaneous Transformation Frequencies per* $10^6$ *Survivors of Cell Types Used in Transformation Assay*

| | Transformation frequency ± s.d. per $10^6$ survivors: BHK 21-Cl 13 (n = 30) | W1–38 (n = 24) | Chang (n = 18) |
|---|---|---|---|
| Untreated | 46·7±6 | 4·3±0·2 | 7·5±2·0 |
| DMSO | 46·7±7 | 4·8±1·3 | 9·2±2·4 |
| S9 mix | 50·2±7 | 4·7±1·5 | 8·9±2·6 |
| DMSO+S9 mix | 49·0±7 | 4·6±1·7 | 9·4±4·3 |
| Water | 48·5±8 | 4·7±1·6 | 9·2±4·0 |
| Water+S9 mix | 50·7±15 | 4·8±1·7 | 8·9±2·7 |

indicate that the species or organ of origin of the tester cells is of no significance in this assay, and that one cell type offers no great predictive advantage over another. Since each cell type was similarly effective in determining carcinogenic potential, there seems to be little need to use more than one cell type in the assay.

Chang cells were used as a replacement for W1-38 when the latter were lost. The last 17 compounds in the study were tested with Chang cells, including 4 compounds previously tested with W1-38. The use of Chang cells did not appear to alter the accuracy of the test and, although they are suspected of being contaminated with HeLa cells (Lavappa *et al.*, 1976), very few negative-control Chang cells were capable of growth in semi-solid agar (Table III.2). Since it is difficult to prove that compounds are non-carcinogenic, the false-positive results may be correct. Alternatively since the mechanism of *in vitro* transformation is obscure and may be altered from that occurring *in vivo*, the false-positive results may be due to unknown causes. It has been suggested that the primary event in carcinogenesis is somatic mutation (Boveri, 1914; Bauer, 1928; Burdett, 1955; Brookes and Lawley, 1964; Miller and Miller 1971; Ames *et al.*, 1973; Malling and Chu, 1974). However, although many carcinogens have been shown to react with DNA, they also react with other cellular macromolecules and it is not known which is the critical target in carcinogenesis. Other observations on cell mutation (Harris, 1974 *a*, *b*) and transformation (Weinstein *et al.*, 1975) suggest that, while the primary event in malignant transformation may be recessive mutation, other changes, mutational or otherwise, are required for a cell and its progeny to be able to progress to a tumour.

A comparison of the data from mammalian cell transformation with those from bacterial mutation reveals that 106 compounds are correctly identified by both tests. Of the 14 remaining compounds 12 are identified correctly by one of the tests (7 by transformation and 5 by bacterial mutation). These results are summarized in Table VI. It appears, therefore, that this transformation assay, in general, is no better than Salmonella or other bacteria in detecting carcinogens. The work of Huberman *et al.*, (1976) and Bouck and Di Mayorca (1976) suggests that *in vitro* transformation is the result of a mutagenic event.

Without additional metabolic activation by rat postmitochondrial supernatant (S9 mix), very few carcinogens transformed hamster or human cells in the period of incubation used. It has been shown that most carcinogens require metabolic activation, and that this may be carried out *in vitro* by using liver homogenates, or metabolically competent feeder layers of mammalian cells, when the test cell has no intrinsic metabolic capability (Malling, 1971; Beije and Hultin, 1971; Miller and Miller, 1971; Garner *et al.*, 1972; Grover *et al.*, 1972; Magee and Farber, 1962; Ames *et al.*, 1973; Huberman and Sachs, 1974; Frantz and Malling, 1975; Huberman, 1975).

Human cells, uninfected with virus, have been found difficult to transform *in vitro* with chemical carcinogens. Chemical transformation of human cells has generally been of genetically abnormal or tumorous or repair-deficient cells (Benedict *et al.*, 1975; Igel *et al.*, 1975; Rhim *et al.*, 1975 *a*, *b*; Shimada *et al.*, 1976). However, Benedict *et al.* (1975), Igel *et al.* (1975) and Kakunaga (1976) have reported *in vitro* transformation of virally uninfected human cells of non-tumorous origin.

In the present study, although both the human cell types used have a detectable level of spontaneous transformation, few carcinogens caused transformation without auxiliary metabolism by rat liver homogenate. The frequency of transformation by carcinogens was, even with activation, 10-fold lower than in BHK cells. The resistance of human cells to transformation by chemicals is possibly due to their being less metabolically active in culture than rodent cells, and less able to bind metabolites to macromolecules (Diamond *et al.*, 1967; Kuroki and Heidelberger, 1972; Marquardt and Heidelberger, 1972; Huberman and Sachs, 1974).

### REFERENCES

AMES, B. N., DURSTON, W. E., YAMASAKI, E. & LEE, F. D. (1973) Carcinogens are Mutagens: a Simple Test System Combining Liver Homogenates for Activation and Bacteria for Detection. *Proc. natn. Acad. Sci. U.S.A.*, **70**, 2281.

AMES, B. N., MCCANN, J. & YAMASAKI, E. (1975) Methods for Detecting Carcinogens and Mutagens with the *Salmonella*/Mammalian Microsome Mutagenicity Test. *Mutation Res.*, **31**, 347.

BAUER, K. H. (1928) Mutations Theorie der Geschwulst-Entstehung. Ubergang von Korperzellen In. *Geschwulstzellen durch Gen-Anderung.* Berlin: J. Springer.

BEIJE, B. & HULTIN, T. (1971) Oxidation and Protein Binding of Aromatic Amines by Rat Liver Microsomes. *Chem. biol. Interact.*, **3**, 321.

BENEDICT, W. F., JONES, P. A., LANG, W. E., IGEL, H. J. & FREEMAN, A. E. (1975) Characterisation of Human Cells Transformed *in vitro* by Urethane. *Nature*, **256**, 322.

BOUCK, N. & DI MAYORCA, G. (1976) Somatic Mutation as the Basis for Malignant Transformation of BHK Cells by Chemical Carcinogens. *Nature*, **264**, 722.

BOVERI, T. (1914) *Zur Frage der Entstehung maligner Tumoren.* Jena: Gustav Fischer.

BROOKES, P. & LAWLEY, P. D. (1964) Evidence for the Binding of Polynuclear Aromatic Hydrocarbons to the Nucleic Acids of Mouse Skin: Relation between Carcinogenic Power of Hydrocarbons and their Binding to Deoxyribonucleic Acid. *Nature*, **202**, 781.

BURDETTE, W. J. (1955) The Significance of Mutation in Relation to the Origin of Tumours: a Review. *Cancer Res.*, **15**, 201.

DIAMOND, L., DEFENDI, V. & BROOKES, P. (1967) The Interaction of 7,12-dimethylbenz(a)anthracene with Cells Sensitive and Resistant to Toxicity Induced by this Carcinogen. *Cancer Res.*, **27**, 890.

DI MAYORCA, G., GREENBLATT, M., TRAUTHEN, T., SOLLER, A. & GIORDANO, R. (1973) Malignant Transformation of BHK21 Clone 13 Cells *in vitro* by Nitrosamines a Conditional State. *Proc. natn. Acad. Sci. U.S.A.*, **70**, 46.

DI PAOLO. J. A. (1974) Quantitative Aspects of *in vitro* Chemical Carcinogenesis. *Biochem. Dis.*, **4**, 433.

DI PAOLO, J. A. (1974) Quantitative Aspects of *in vitro* Chemical Carcinogenesis. In *Chemical Carcinogenesis* (Part b). Ed. P. O. Ts'O and J. A. Di Paolo. New York: Marcel Dekker Inc. p. 443.

FRANTZ, C. N. & MALLING, H. V. (1975) The Quantitative Microsomal Mutagenesis Assay Method. *Mutation Res.*, **31**, 365.

GARNER, R. C., MILLER, E. C. & MILLER, J. A. (1972) Liver Microsomal Metabolism of aflatoxin B1 to a Reactive Derivative Toxic to *Salmonella typhimurium* TA 1530. *Cancer Res.* **32**, 2058.

GROVER, P. L., HEWER, A. & SIMS, P. (1972) Formation of K-region Epoxides as Microsomal Metabolites of Pyrene and Benzo(a)pyrene. *Biochem. Pharmacol.*, **21**, 2713.

HARRIS, M. (1974*a*) Mechanisms of *de novo* Variation in Mammalian Cell Cultures In *Somatic Cell Hybridization.* Eds. R. L. Davidson and Felix de la Cruz. New York: Raven Press. p. 221.

HARRIS, M. (1974*b*) Induction of Variants by Nitrosoguanidine in Diploid and Tetraploid Lines of Chinese Hamster Cells. In. *Chemical Carcinogenesis* (*part b*). Ed. P. O. Ts'O, and J. A. Di Paolo. New York: Marcel Dekker Inc. p. 575.

HEIDELBERGER, C. (1973) Chemical Oncogenesis in Culture. *Adv. Cancer Res.*, **18**, 317.

HEIDELBERGER, C. (1973) Current Trends in Chemical Carcinogenesis. *Fed. Proc.*, **32**, 2154.

HEIDELBERGER, C. (1975) Chemical Carcinogenesis. *Ann. Rev. Biochem.*, **44**, 79.

HUBERMAN, E. (1975) Mammalian Cell Transformation and Cell Mediated Mutagenesis by Carcinogenic Polycyclic Hydrocarbons. *Mutation Res.*, **29**, 285.

HUBERMAN, E., MAGER, R. & SACHS, L. (1976) Mutagenesis and Transformation of Normal Cells by Chemical Carcinogens. *Nature*, **264**, 360.

HUBERMAN, E. & SACHS, L. (1974) Metabolism of the Carcinogenic Hydrocarbon Benzo(a)pyrene in Human Fibroblast and Epithelial Cells. *Int. J. Cancer*, **11**, 412.

HUBERMAN, E. & SACHS, L. (1974) Cell-mediated Mutagenesis of Mammalian Cells with Chemical Carcinogens. *Int. J. Cancer*, **13**, 326.

IGEL, H. J., FREEMAN, A. E., SPIEWAK, J. E. & KLEINFELD, K. L. (1975) Carcinogenesis *in vitro* II Chemical Transformation of Diploid Human Cell Cultures: a Rare Event. *In vitro*, **11**, 117.

KAKUNAGA, T. (1977) The Transformation of Human Diploid Cells by Chemical Carcinogens In *Origins of Human Cancer*. Cold Spring Harbor Conferences on Cell Proliferation. **4**, 1537.

KUROKI, T. & HEIDELBERGER, C. (1972) Determination of the b-protein in Transformable Cells and Transformed Cells in Culture. *Biochemistry*, **11**, 2116.

LAVAPPA, K. S., MACY, M. L. & SHANNON, J. E. (1976) Examination of ATCC Stocks for HeLa Marker Chromosomes in Human Cell Lines. *Nature*, **259**, 211.

MARQUARDT, H. & HEIDELBERGER, C. (1972) Influence of "Feeder Cells" and Inducers and Inhibitors of Microsomal Mixed Function Oxidase on Hydrocarbon-induced Malignant Transformation of Cells Derived from C3H Mouse Prostate. *Cancer Res.*, **32**, 721.

MAGEE, P. N. & FARBER, E. (1962) Methylation of Rat Liver Nucleic Acids by Dimethylnitrosamine *in vivo*. *Biochem. J.*, **83**, 114.

MALLING, H. V. (1971) Dimethylnitrosamine: Formation of Mutagenic Compounds by Interaction with Mouse Liver Microsomes. *Mutation Res.*, **13**, 424.

MALLING, H. V. & CHU, E. H. Y. (1974) Development of Mutational Model Systems for Study of Carcinogenesis. In *Chemical Carcinogenesis* (*Part B*). Eds. P. O. Ts'O and J. A. Di Paolo. New York 1974: Marcell Dekker Inc. p. 545.

MILLER, E. C. & MILLER, J. A. (1971) The Mutagenicity of Chemical Carcinogens Correlations, Problems and Interpretation. In *Chemical Mutagens: Principles and Methods for Detection, Vol.* 1 Ed. A. Hollaender. New York: Plenum Press. p. 83.

MISHRA, N. K. & DI MAYORCA, G. (1974) *In vitro* Malignant Transformation of Cells by Chemical Carcinogens. *Biochim. biophys. Acta.*, **355**, 205.

RHIM, J. S., KIM, C. M., ARNSTEIN, P., HUEBNER, R. J., WEISBURGER, E. K. & NELSON-REES, W. A. (1975*a*) Transformation of Human Osteosarcoma Cells by a Chemical Carcinogen. *J. natn. Cancer Inst.*, **55**, 1291.

RHIM, J. S., PARK, D. K., ARNSTEIN, P., HUEBNER, R. J., WEISBURGER, E. K. & NELSON-REES, W. A. (1975*b*) Transformation of Human Cells in Culture by N-methyl-N′-nitro-N-nitrosoguanidine. *Nature*, **256**, 751.

SHIMADA, H., SHIBUTA, H. & YOSHIKAWA, M. (1976) Transformation of Tissue Cultured Xeroderma Pigmentosum Fibroblasts by Treatment with N-methyl-N′-nitro-N-nitrosoguanidine. *Nature*, **264**, 547.

STYLES, J. A. (1977) A Method for Detecting Carcinogenic Organic Chemicals using Mammalian Cells in Culture. *Br. J. Cancer*, **36**, 558.

WEINSTEIN, B., YAMAGUCHI, N., GEBERT, R. & KAIGHN, M. E. (1975) Use of Epithelial Cell Cultures for Studies on the Mechanism of Transformation by Chemical Carcinogens. *In vitro*, **11**, 130.